# 

**Published:** 1846-02

**Authors:** 


### WESTERN JOURNAL

OF

### MEDICINE AND SURGERY.

### EDITORS.

---

DANIEL DRAKE, M.D.,

PROFESSOR OF PATHOLOGY AND THE PRACTICE OF MEDICINE IN THE MEDICAL INSTITUTE OF LOUISVILLE:

LUNSFORD P. YANDELL, M.D.,

PROFESSOR OF CHEMISTRY AND PHARMACY IN THE SAME:

THOMAS W. COLESCOTT, M.D.

### BOOKS RECEIVED.

In addition to the works already noticed, we have received from Wiley & Putnam, through Messrs. Maxwell and Prescott of this city, the following works:

*Gray's Botanical Text Book*, a neat duodecimo volume, illustrated with numerous wood cuts, of 500 pages. This, we believe, is esteemed the best elementary work on botany extant in our country.

*Newnham's Human Magnetism.*—This is an attempt to show the utility of the application of mesmerism to the relief of human suffering. We confess our want of faith in its efficacy, but nevertheless shall give the author a hearing. We shall look into his "dispassionate inquiry" in a future number. Meantime, those who are curious on the subject will find it discussed in this work by one who is a thorough convert to its mysteries and its magic.

*Fowne's Prize Essay.*—This is a charming work; we have read it with the greatest pleasure. It combines every element of interest —a simple, lucid style, scientific details novel to all but the professed chemist, and views of the arrangement and constitution of matter which fill the mind with admiration and delight. The subject is "Chemistry as exemplifying the Wisdom and Benevolence of God." A more fascinating volume no one cold wish to possess.

*Lyell's Travels in North America.*—This is a most captivating book of travels, abounding in just and accurate observations, liberal views, and scientific information interesting to every class of readers, especially those who have a taste for geology. Mr. Lyell is one of the most gifted and learned foreigners who has traversed our great continent, and his remarks on its configuration and structure, its fossils and its animals, its rivers, lakes, and estuaries, its institutions and people, will be found worthy of his high character. We hope to find room, during the summer, for an extended notice of his Travels.

To our friends, Lea & Blanchard, we are indebted for several works of great value, most of which we shall be unable to notice, at any great length, in our present number. These enterprising publishers have issued new editions of *Meigs' Velpeau's Midwifery*, and *Churchill's System of Midwifery*, which standard works will be found at the bookstores of Maxwell and Prescott.

*Durlacher on Corns, Bunions, Diseases of the Nails, and general management of the Feet.*—This is the first treatise on these ailments, so far as we are informed, that has issued from the American press. It is from the pen of the '*Surgeon Chiropodist*, by special appointment, to the Queen,' and appears to be scientific. We shall notice it hereafter. It is from the press of Lea & Blanchard, who take care never to send forth anything trashy.

### *Receipts of Medical Journal for January*, 1846.

| | |
|---|---|
| Dr. J. S. Athon, Charlestown, Ind., | $5 00 |
| Dr. C. White, Paoli, Ind., | 10 00 |
| Dr. Desha, Georgetown, Ky., | 2 50 |
| Dr. R. Hamilton Morristown, Ohio, | 5 00 |
| Dr. J. F. Fleming, Elizaville, Ky., | 5 00 |
| Dr. J. Barnes, McMinville, Tenn., | 5 00 |
| Dr. J. Roberts, Montezuma, Ind., | 5 00 |
| Dr. A. Brunson, Richland, Ark., | 10 00 |
| Dr. McLain, Carthage, Tenn., | 5 00 |
| Dr. D. Love, Jonesboro', Ill., | 5 00 |
| Dr. R L. Wilkinson, New Haven, Ky., | 5 00 |
| Dr. J. Slevens, Portland Mills, Ind., | 5 00 |
| Dr. S. Wort, Brownstown, Ind., | 7 00 |
| Dr. T. P. Albertson, Brownstown, Ind., | 5 00 |
| Dr. L. E. Laird, Ripley, Miss., | 5 00 |
| Dr. N. D. Thompkins' Martinsville, Ind., | 5 00 |
| Dr. H. Hard, Plymouth, Ind., | 5 00 |
| Dr. S. M'Cullough, Plymouth, Ind. | 5 00 |
| Dr. W. Riker, do. do., | 5 00 |
| Dr. W. Priest, do. do., | 5 00 |
| Dr. A. J. Menich, Freedom, Ind., | 5 00 |
| Drs. Moore & Short, Hartford, Ky., | 5 00 |
| Dr. J. W. Beauchamp, Edmonton, Ky. | 5 00 |
| Dr. Clapp, New Albany, Ind., | 5 00 |
| Dr. S. Jackson, Columbus, Ind. | 5 00 |
| Dr. J. Smick, Darwin, Ill, | 10 00 |
| Dr. D. Patton, Finley, Ohio, | 10 00 |
| Dr. S. B. McClurken, Halsellville, S. C., | 5 00 |
| Dr. S. Christy, Farmington, Ill., | 5 00 |
| Dr. J. Davisson, Petersburg, Ind., | 5 00 |
| Drs. Spencer & Groves, Franklin, Ky., | 5 00 |
| Dr. S. B. Robinson, Murfreesboro', Tenn., | 5 00 |
| Dr. B. Burwell, Buffalo, N. Y. | 25 00 |
| Dr. J. S. Dodge, Cincinnati, Ohio., | 26 00 |
| Dr. E. Jones, Mt. Pleasant, Ohio.. | 5 00 |
| Dr. J. M. Morris, Somerset, Ohio, | 5 00 |
| Dr. D. Cox, New Paris, Ohio, | 5 00 |
| Dr. A. Beasley, Ripley, Ohio, | 10 00 |
| Dr. D. Dayton, South Bend, Ind., | 5 00 |
| Dr. J. C. Helm, Greenville, Ind., | 5 00 |
| Dr. T. R. O'Hara, Carlisle, Ind., | 5 00 |
| Dr. J. McFarland, Lafayette, Ind., | 10 00 |
| Dr. J. N. Crooks, Rockport, Ind. | 5 00 |
| Dr. J. Carr, Princeton, Ky., | 5 00 |
| Dr. J. Whitlock, Bellview, Ky., | 10 00 |
| Dr. D. G. Dedman, Lawrenceburg, Ky., | 10 00 |
| Dr. J. Westerfield, Glasgow, Ky., | 5 00 |
| Dr. J. H. Tobin, Burksville, Ky., | 10 00 |
| Dr. M. Ray, Liberty, Ky., | 5 00 |
| Dr. J. N. Hodges, Tompkinsville, Ky., | 5 00 |

### TRAVELING AGENTS FOR THE WESTERN JOURNAL OF MEDICINE AND SURGERY.

JNO. F. JOHNSTON and JAS. H. STEVENSON, Kentucky—C. W. JAMES, Ohio, Indiana, Illinois, Missouri, and Michigan—GEO. C. BRETT, Tennessee—WM. H. WELD & SON, Mississippi and Louisiana.

The WESTERN JOURNAL OF MEDICINE and SURGERY is published monthly by the undersigned, at the corner of Main and Fifth streets, Louisville, at $5 per annum, payable in advance. Each number contains 96 pages, making two volumes in the year of more than 550 pages.

Contributions to its pages by the Physicians of the Valley of the Mississippi, addressed to the Editors, are respectfully solicited.

Letters on business, to be addressed, postage paid, to the publishers.

*January*, 1844. PRENTICE & WEISSINGER

